# Surface Anatomy of the Thorax

**Published:** 1901-07-20

**Authors:** Seymour Taylor

**Affiliations:** Physician to the Hospital


### Hospital Clinics and Medical Progress.

### SURFACE ANATOMY OF THE THORAX.

(*Continued.*)

Abstract of a Clinical Lecture delivered at the West London Hospital in May 1901, by Seymour Taylor, M.D., F.R.C.P., Physician to the Hospital.

Let us now come down to the thorax itself. Still keeping in the middle line we pass our fingers on to the sternal notch, where one may sometimes detect a pulsation or aneurysm of the aorta, since the aorta itself is not above an inch and a quarter from the notch. We notice next the ridge between the manubrium and the gladiolus of the sternum, a ridge which is subcutaneous, easily felt even in the fattest subjects, and always constant in its locality. It marks the mid line of junction of the second costal cartilages to the sternum. At the end of the gladiolus we may easily feel the xiphoid cartilage. It varies in length considerably and it varies in position. The most important variation in position, however, is its alteration in plane. It may be perfectly flat and continuous with the surface of the sternum, or on the other hand it may project considerably upwards. If it is found to project upwards to any great degree, it is very fair evidence that the man has at one period of his life been very corpulent, and that he has lately lost flesh; for the cartilage itself is part of the arc of the belly-curvature, and emaciation having occurred, the belly may fall in, leaving the cartilage somewhat erect showing the ruins as it were of an arch. This observation is not my own; the credit of it is due to Ord; but that it is absolutely correct as a clinical fact I have been able to verify times out of number.

Another constant landmark on the male chest is the nipple. The nipple is on the costal end of the fourth rib, rather to its lower edge than its upper. This is so constant a landmark in the male that I use it as a point of measurement in recording adventitious sounds, especially those of the lungs, which may be detected by exploration, whether it be by the stethoscope or by percussion. I like not the nomenclature which obtains when discussing the region of the chest. Such terms as inframammary, supra- and infra- clavicular are somewhat cumbersome. The way I record any physical sign occurring in the lungs or pleura is to regard the nipple as the centre of a clock-dial, and measure with the tape from this landmark the spot where these adventitious sounds are heard. For instance, if I hear cavernous breathing in the apex of the right lung, immediately above the nipple, I record in my notebook, "Cavernous breathing; right nipple; 12 o'clock." If, again, I hear friction in the axillary line, say 4 inches from the right nipple in the direction of 9 o'clock on the clock-face, I record, "Friction; right nipple; 4 inches; 9 o'clock." I find this a quick and easy way of recording physical signs, and I adopt a similar method when I explore the back of the chest, taking the spines of one of the vertebræ as the centre of my dial. I do not say that this is an absolutely perfect way of recording symptoms, but in the stress of seeing a large number of patients I am convinced that you will find it a very rapid method.

On turning to the dorsal surface of the chest I would point out to you that the scapulæ run from the second ribs down to the seventh. I would also draw your attention to the fact that amongst the many other important landmarks which you must know, there is one which is often overlooked or forgotten. It is a triangular space at the lower part of the vertebral border of the scapula in which the lung is practically subcutaneous, being covered by no muscles to deaden respiratory sounds. The base of this triangular space may be represented by the lower third of the vertebral border of the scapula. Its lower side is formed by the upper border of the latissimus dorsi, and its upper border by the lower border of the trapezius. In this space one can easily detect adventitious sounds which may have generated at the lower lobe of the lung.

The great fissure of the lung is an important line, and should be mapped out accurately. I have been accustomed to call it "Fowler's line" in my class since Dr. Kingston Fowler drew attention to the frequency of the deposit of tubercular disease along this fissure. If the arm is placed across the chest, and the hand made to grasp the opposite shoulder, a line drawn from the second dorsal spine downwards and outwards to the inferior angle of the scapula, and then forwards to the lower end of the sternum would fairly accurately mark out the course of the great fissure separating the upper from the lower lobes of the lungs. Its importance is, as I have told you, great by reason of the frequency of tubercular deposit along this fissure.

There is one point in conclusion to which I would like to draw your attention, and that is the extent of lung tissue that exists above the planes of the clavicles. The apex of the right lung extends up into the neck one and a half inches, and on the left side it runs up not quite so far—only about an inch. This part of the lung is covered by the scalenii muscles and the pleura investing it is strengthened by the deep fascia of the neck which envelops the scalenii muscle, and is prolonged on to the pleura. A knowledge of this extension of the lung into the neck is important as bearing upon wounds in the neck, whether inflicted by knife or by bullet. It is also important inasmuch as it will account for a deal of the pain which occurs in the neck in patients who are the subject of tuberculous deposits.

